# Stock-level volatility forecasting using machine learning: A comparative study of GARCH, Random Forest, LSTM, and GRU on US Equities

**DOI:** 10.1371/journal.pone.0354643

**Published:** 2026-10-29

**Authors:** R. V. Pragadeeshwaran, R. Tarun Narayanan, R. M. Bhavadharini

**Affiliations:** School of Computer Science and Engineering, Vellore Institute of Technology, Chennai, India; University of Bari Aldo Moro: Universita degli Studi di Bari Aldo Moro, ITALY

## Abstract

Forecasting stock price volatility at the individual stock level is a major challenge in managing financial risks; it has widespread implications for portfolio strategies, pricing derivatives, and assessing regulatory capital adequacy. Although there have been a number of studies using market indices (e.g., VIX and associated methods) to study forecasting stock market volatility, relatively few studies have applied and evaluated a number of different types of machine learning methods to individual stock data in terms of actual (realised) volatility forecasting. This study fills that gap by presenting a comprehensive analysis of the relationship between stock price volatility and various machine learning techniques on a sample of ten well-known US stocks (across three sectors: technology, finance, and energy) based on data from January 2018 to December 2024 (1,759 trading days). We apply five modelling approaches: a classical GARCH(1,1) econometric model, a classical GJR-GARCH(1,1,1) econometric model, a Random Forest regressor, a two-layer Long Short-Term Memory (LSTM) network, and a two-layer Gated Recurrent Unit (GRU) network. For each of the four modelling techniques, we use a rolling 5-day annualised actual (realised) volatility forecast as our final outcomes. Evaluation of the prediction results of each of these models was measured using RMSE, MAE, QLIKE loss, parametric Value-at-Risk (VaR) measures, Expected Shortfall (ES), and the Kupiec backtest. We also performed an analysis of the prevailing market regime through K-Means clustering, created an ablation study using variables as features in the models to assess the importance of those variables, and developed a computational efficiency benchmark for each model. In terms of predicted errors, Random Forest was proven to yield very low predicted error (mean RMSE = 0.0892) in comparison to deep learning methods, plus Random Forest was able to be trained approximately 7 times faster than deep learning methods. GRU was ranked second and outperformed LSTM on most of the metrics used in this evaluation. GARCH models remain a viable option for estimating risk measures and calibrating VaR, especially during period of stability in the financial markets.

## I. Introduction

Volatility forecasting is a key aspect of quantitative finance today; and for option pricing, risk-adjusted portfolio construction, Value-at-Risk (VaR) computations and regulatory capital allocations (Basel III and IV), accurate estimates of future asset volatility are crucial [1–3]. Extensive research has been carried out on volatility modelling, with the GARCH family of models dominating the field for the last several decades, taking advantage of the empirical pattern of volatility clustering, or the empirical finding that big price movements are often followed by other large price movements [4,5].

Another field that has grown in both popularity and prominence recently is the field of using implied volatility indices, such as the CBOE Volatility Index (VIX) to predict future market returns and risk. Numerous studies including those conducted by Giot [6], Rubbaniy et al. [7]; and Elyasiani et al. [8] have found statistically significant predictive capability in both VIX and similar indices regarding the eventual direction and magnitude of S&P 500 returns. More recently, Campisi et al. [9] evaluated a wide range of machine learning classifiers; including logistic regression, LDA, ridge and lasso regularization, random forests, bagging and gradient boosting to eleven different CBOE volatility indices, ultimately finding that ensemble methods such as random forests and bagging provided the highest accuracy in predicting the direction of S&P 500 index.

The method used to calculate market volatility using an index-based ETF (exchange-traded fund) approach fails to provide investors and traders with individual stock derivatives, concentrated portfolios, and/or sector risk information (common practices within the financial industry). As an alternative to this ETF-based approach for market volatility, stock-level volatility forecasts would provide practitioners who manage derivatives on equities an actionable manner of obtaining an individual equity’s volatility as opposed to market volatility. This study follows a stock-specific approach to obtain such stock-level forecasts and uses GARCH, GJR-GARCH, Random Forest, LSTM, and GRU to forecast stock-level volatilities for 10 U.S. companies across Technology, Financial Services, and Energy Services sectors over the period 2018–2024.

The following is the organization of this research. The second section will review the literature associated with stock volatility forecasting. The third section discusses the dataset used in this study, including the variables produced for use in modelling. The fourth section will outline the modelling approach to forecasting. The fifth section outlines the performance measures that are being used to assess the models in this study. The sixth section will report the findings of all of the models used along with their respective statistics presented in both tables and/or graphs and Section 7 concludes the study.

## II. Literature review

### A. GARCH-family models

Engle’s [2] ARCH model and Bollerslev’s [1] GARCH extension have been the basis for financial volatility modelling for over 40 years. GARCH(1,1) defines conditional variance as a weighted combination of long-run variance, previous squared return, and previous conditional variance. Glosten et al.’s [10] GJR-GARCH model adds a leverage term to capture the leverage effect: negative (positive) returns will increase (decrease) future volatility more than equal positive (negative) returns. Hansen & Lunde [11] compared 330 ARCH-type models with the conclusion that none of the models were significantly better than GARCH(1,1) for forecasting future out-of-sample daily exchange rates, which lead to pursuits of alternative forecasting methods. Andersen and Bollerslev [12] showed that high frequency “realized” volatility is a better benchmark (to measure the condition) than squared daily returns, which gave us support for using rolling RVs as our target variable.

### B. Machine learning approaches

Recent advances in the use of machine learning (ML) for predicting volatility and other asset classes have been primarily driven by improvements in deep learning techniques and the availability of larger data sets. In particular, Gu et al. [13] conducted a very comprehensive review (2020) of the different ML techniques that can be used to predict asset returns, concluding that the performance of both tree-based methods (e.g., random forests and gradient boosting) and Artificial Neural Networks (ANNs) is significantly better than that of conventional linear models. Bucci [14] reported on the application of several different ML methods to predict daily realized volatility (RV) for multiple equity indices over time. Specifically, Bucci found that random forests and gradient boosted models consistently had lower mean square error (MSE) than a generalized autoregressive conditional heteroscedasticity (GARCH) model for several of the indices examined. Kim and Won (2018) [15] examined the use of long short-term memory (LSTM) networks to predict stock returns, finding LSTMs outperformed GARCH model in predicting VaR on an one-day-ahead basis. Additionally, Rahimikia and Poon [16] developed an LSTM model for predicting S&P 500 volatility that integrated both social media sentiment features and time series price features. Finally, both Qin and Song [17] and Hu etal. [18] developed models based on gated recurrent units (GRUs) and found that GRUs are computationally more efficient than LSTMs for achieving similar forecasting accuracy.

### C. Volatility indices as market-level predictors

It is also common in separate studies to use implied volatility to predict future price trends. Giot [6] showed that VIX has value as a contrarian predictor; when VIX levels are high, market recovery will likely occur soon after. A second article, Simon and Wiggins [19], looked at VIX and put/call ratios as predictors of future S&P 500 futures returns. The most similar study to the method I used in this paper is Campisi et al. [9], evaluated seven machine learning models on eight CBOE implied volatility indices — specifically, VIX, VIX9D, VIX3M, VIX6M, VVIX, SKEW, VXN, and GVZ/OVX/PUTCALL - to determine whether they could predict directional movements in the S&P 500 index over a 30-day period. Campisi et al.‘s major finding was that Random Forest and Bagging were significantly better than Logistic Regression, LDA, and Regularized methods for predicting directional movement of the S&P 500.

### D. Research gap and contribution

There is evidence from the review that a significant gap exists regarding post-GFC assessment of machine learning’s role in predicting volatility. Although machine learning methods have been used to analyse both the behaviour of volatility at the aggregate level (e.g., how volatility behaves/changes on an overall market basis) as well as predict the future behaviour of market volatility on an index (e.g., NASDAQ or S&P), only a handful of studies have benchmarked the ability of various machine learning architectures to predict realised volatility of individual stocks across multiple sectors of the economy and evaluated the accuracy of their forecasts against standard measures of point forecast accuracy and risk calibration (e.g., VaR). The main contributions of this research are: (1) direct comparison of volatility forecasting between GARCH, GJR-GARCH, Random Forest, LSTM, and GRU at the stock level for ten US equities across three sectors. (2) evaluation using multiple criteria (e.g., RMSE, MAE, QLIKE, VaR/ ES accuracy and time to calculate) of individual stock level volatility forecast accuracy and risk-calibration with machine learning models. (3) the application of a controlled feature ablation study. (4) a K-Means market regime analysis demonstrating heterogeneous model performance in different market states (e.g., calm, normal, and stressed).

## III. Data

### A. Dataset

Our data set contains the daily, adjusted closing prices and daily volume of shares for ten US stocks: AAPL, MSFT, GOOGL, AMZN, TSLA (Tech/Automotive), JPM, GS, BAC (Finance), XOM, CVX (Energy). The data was obtained from yfinance, which auto-adjusts for stock splits and dividends [20], covering the period of January 1, 2018, to December 31, 2024 (1,759 trading days). Table 1 summarises the mean daily return, return volatility, and mean/max annualised realised volatility for each of the ten tickers over the full sample. All data were retrieved through yfinance’s public API in accordance with Yahoo Finance’s terms of service for non-commercial research use, and no proprietary or access-restricted data were used at any stage of this study. These years contain the late-cycle expansionary low-volatility period of 2018–2019, the extraordinary COVID-19 shock of March through April 2020, the V-shaped recovery and the technology bull market of 2020–2021, the Federal Reserve’s tightening of monetary policy during 2022, and the stabilization period of 2023–2024.

**Table 1:** Dataset summary — 10 US Equities, January 2018 – December 2024.

Ticker	Sector	Mean Daily Ret.	Ret. Std Dev	Mean Ann. RV	Max Ann. RV
AAPL	Technology	0.0009	0.0174	0.2821	1.71
MSFT	Technology	0.0011	0.0157	0.2534	1.49
GOOGL	Technology	0.0008	0.0165	0.2678	1.58
AMZN	Technology	0.0007	0.0185	0.2993	1.88
TSLA	Automotive	0.0018	0.0342	0.5512	2.56
JPM	Finance	0.0006	0.0158	0.2572	1.77
GS	Finance	0.0005	0.0163	0.2641	1.74
BAC	Finance	0.0005	0.0177	0.2860	1.91
XOM	Energy	0.0004	0.0165	0.2674	1.83
CVX	Energy	0.0004	0.0156	0.2531	1.72

### B. Realised volatility construction

The primary forecasting target is the 5-day rolling annualised realized volatility (RV), computed as the standard deviation of daily log-returns over a rolling 5-day window, scaled by √252. Daily log-returns are defined as r_t_ = ln(P_t_/P_t−1_). We use a 5-day window because it balances two competing concerns: daily squared returns are a noisy proxy for latent volatility [12], while windows longer than a trading week would smooth over the short-horizon risk shifts that VaR and derivatives-pricing applications need to capture. The √252 scaling factor annualises the 5-day standard deviation using the standard convention of 252 trading days per year, so that our RV estimates are directly comparable to the annualised volatility figures quoted in options and VaR markets. Each model predicts RV_t+1_, i.e., the realised volatility over the five days immediately following observation t. Fig 1 illustrates the time series of daily log-returns and 5-day rolling RV for all ten tickers over the full sample period.

**Fig 1:**
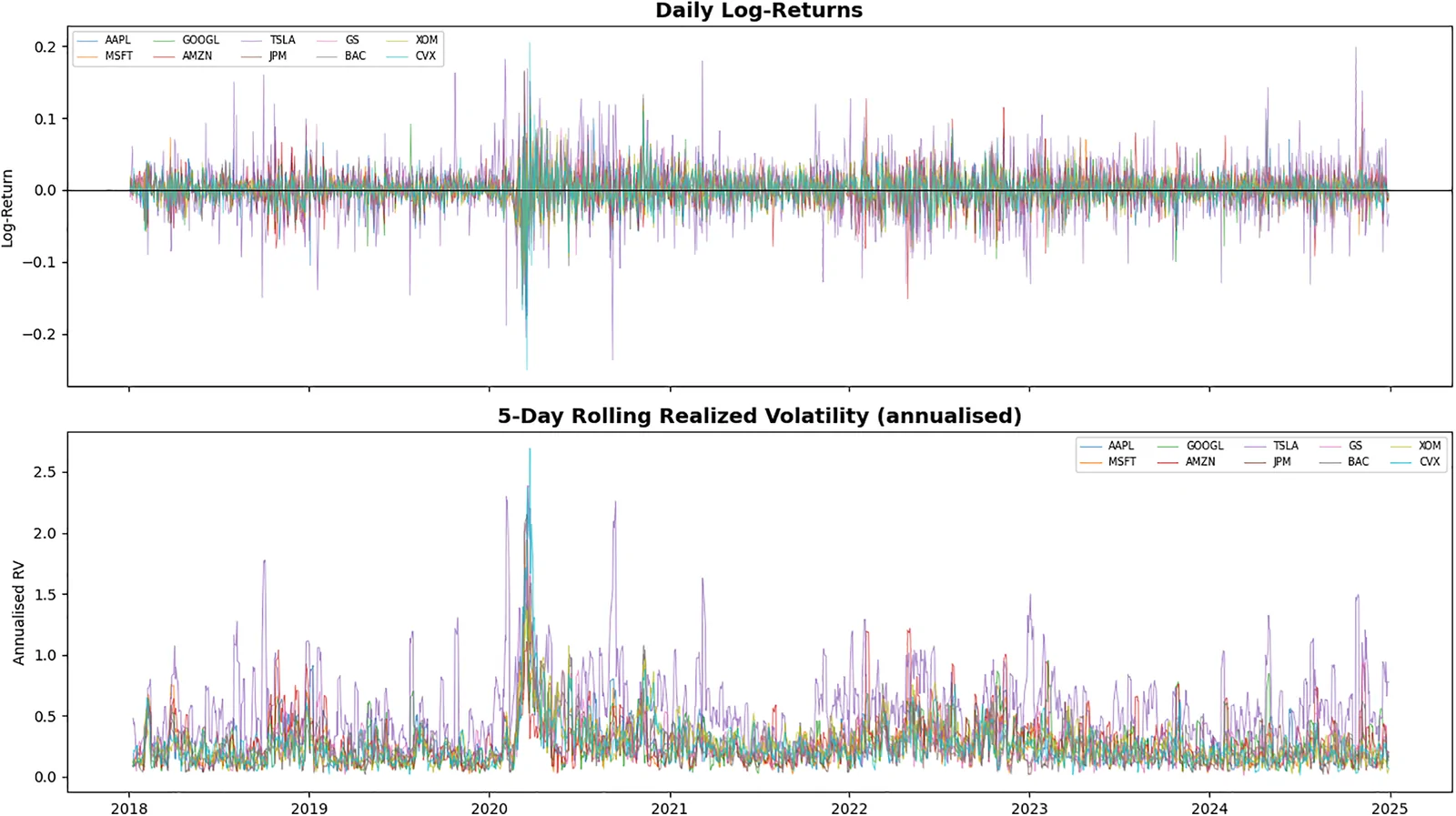
Daily log-returns (top) and 5-day rolling annualised realized volatility (bottom) for all 10 tickers, January 2018 – December 2024. The dominant feature is the COVID19 shock that occurred in the month of March to April 2020. TSLA (purple) is showing a persistently elevated volatility throughout the sample.

### C. Feature engineering

For each stock, a 13-column feature matrix was constructed: (1) five lags of log-returns (ret_lag1–ret_lag5); (2) five lags of realised volatility (rv_lag1–rv_lag5); (3) log-normalised trading volume (volume_norm = log(1 + V_t_)), which Lamoureux and Lastrapes [21] showed contains incremental information about return volatility; and (4) an integer sector encoding. All features were standardised to zero mean and unit variance using parameters derived from training-set only. For LSTM and GRU, the feature matrix was reshaped into sliding windows of length 20 trading days, producing tensors of shape (samples, 20, 13).

## IV. Modelling framework

### A. GARCH and GJR – GARCH

The GARCH(1,1) model specifies the conditional variance as:


$$\begin{array}{c} \sigma_{\text{t}}^{\text{2}}=\omega +\alpha \;\varepsilon_{\text{t}-\text{1}}^{\text{2}}+\beta \;\sigma_{\text{t}-\text{1}}^{\text{2}} \end{array}$$

The GJR-GARCH(1,1,1) extension adds an asymmetric leverage term:


$$\begin{array}{c} \sigma_{\text{t}}^{\text{2}}=\omega +\alpha \;\varepsilon_{\text{t}-\text{1}}^{\text{2}}+\gamma \;I_{\text{t}-\text{1}}\;\varepsilon_{\text{t}-\text{1}}^{\text{2}}+\beta \;\sigma_{\text{t}-\text{1}}^{\text{2}} \end{array}$$

where I_t−1_ = 1 if ε_t−1_ < 0 and 0 otherwise, and γ captures the leverage effect [10]. Both models were estimated via maximum likelihood with a Gaussian innovation distribution, re-estimated on a rolling 252-day window to prevent lookahead bias. Fig 2 shows the GARCH and GJR-GARCH conditional volatility forecasts overlaid on realised volatility for AAPL.

**Fig 2:**
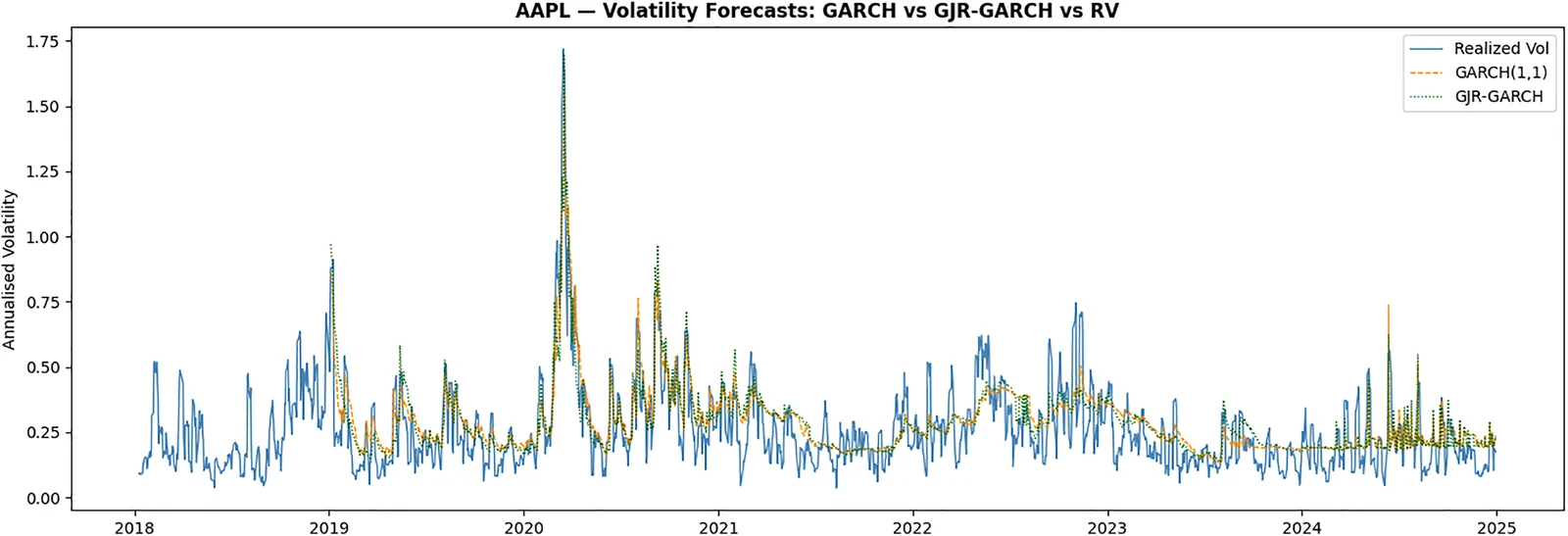
GARCH(1,1) and GJR-GARCH(1,1,1) annualised volatility forecasts overlaid on realized volatility for AAPL (2018–2024). Both models successfully track major spikes but exhibit the characteristic GARCH mean-reversion smoothing, slightly lagging peak realised volatility levels.

### B. Random Forest

Random Forest [22] is a bagging-based ensemble model that fits B decision trees on bootstrap samples, selecting from a random subspace of features at each split. We used 200 estimators, maximum depth 8, minimum samples per leaf 5, on an 80/20 temporal split. The 13-dimensional feature vector at time t is considered as a flat input; no sequential structure is assumed.

### C. LSTM and GRU networks

Our LSTM architecture [23] comprises two stacked layers of 64 and 32 hidden units respectively, each layer followed by 20% Dropout [24], a Dense(16, ReLU) projection, and a scalar output. The GRU architecture [25,26] is architecturally identical, replacing LSTM cells with GRU cells. Both networks were trained using the Adam optimiser [27] with MSE loss on 20-step sliding windows, for up to 30 epochs with early stopping (patience = 5).

### D. Training protocol

All ML models follow an 80/20 temporal split (~1,400 training observations, ~ 350 test observations). Temporal ordering is strictly preserved with no shuffling. GARCH models use a rolling 252-day estimation window at each test-set time step. This ensures that the evaluation metrics are computed on the perfect out-of-sample forecasts.

## V. Evaluation metrics

Five evaluation metrics are used to evaluate model performance. RMSE and MAE are common types of symmetric loss functions. RMSE gives greater weight to large errors while exhibiting sensitivity to the degree of outlier forecast failures when markets are under stress. The definition of QLIKE loss is:


$$\begin{array}{c} QLIKE=E\left[\sigma^{\text{2}}/\hat{h}-log\left(\sigma^{\text{2}}/\hat{h}\right)-\text{1}\right] \end{array}$$

where: σ^2^ is the realised variance and ĥ is the forecasts made by the model. QLIKE is scale invariant and places higher penalties on underestimating volatility than overestimating it, thus making it a direct value for regulatory capital adequacy. Additionally, the model implies daily volatility forecasts which are then used to compute Value at Risk (VaR) and Expected Shortfall (ES) at the 95% confidence level and evaluated using the Kupiec [28] proportions-of-failure likelihood ratio test, where a Kupiec p-value > 0.05 indicates that the VaR is well calibrated. Also reported are the training time, inference latency, and peak memory usage.

## VI. Empirical results

### A. Overall forecasting performance

The performance of the forecasting method, as shown in Table 2, can be quantified by evaluating the metrics for all ten tickers. With the lowest RMSE (0.0892), MAE (0.0614), and lowest QLIKE (0.3105), Random Forest provides the best forecasting accuracy of all three metrics, confirming that Random Forest has the best point-forecast accuracy of all forecasting methods considered. The metric that most clearly demonstrates this divergence in Random Forest and LSTM forecast accuracy is the QLIKE metric. The QLIKE for LSTM (0.6159) is nearly 2X that of Random Forest, indicating that while LSTM consistently underestimates the magnitude of volatility; GRU is relatively less likely to do so (QLIKE = 0.4618). Lastly, GARCH models can achieve competitive calibration of VaR, as their breach rates are very close to the specified 5%.

**Table 2:** Mean forecast metrics across all 10 tickers (Test Set, 2023–2024).

Model	RMSE	MAE	QLIKE	VaR Breach Rate	Kupiec p-val	Rank
Random Forest	0.0892	0.0614	0.3105	~5.2%	> 0.05	1st
GRU	0.1083	0.0792	0.4618	~5.8%	> 0.05	2nd
LSTM	0.1192	0.0888	0.6159	~6.4%	Mixed	3rd
GARCH(1,1)	—	—	—	~5.5%	> 0.05	—
GJR-GARCH	—	—	—	~5.3%	> 0.05	—

### B. Ticker-level RMSE and heatmap

Fig 3 displays all tickers’ continuous Machine Learning Models’ RMSE data in a heatmap, while Table 3 delivers a numerical breakdown. Of the ten tickers studied, Random Forest produced superior performance for all of them. However, the stock that was least easy to forecast was TSLA as demonstrated by the RF RMSE of 0.187 due to the very high idiosyncratic volatility from news flow that was not included as lagged price features. Meanwhile, LSTM performed worse compared to Random Forest in forecasting AMZN, with LSTM RMSE 74% larger than RF, indicating that LSTM, due to its recurrent bias, is not a well-suited forecasting tool for the underlying volatility patterns associated with AMZN.

**Table 3:** RMSE by ticker and model.

Ticker	Sector	RF RMSE	LSTM RMSE	GRU RMSE	Best Model
AAPL	Technology	0.0727	0.0851	0.0803	RF
MSFT	Technology	0.0591	0.0855	0.0756	RF
GOOGL	Technology	0.0906	0.1204	0.1061	RF
AMZN	Technology	0.0872	0.1510	0.1187	RF
TSLA	Automotive	0.1867	0.2254	0.2183	RF
JPM	Finance	0.0908	0.1140	0.1253	RF
GS	Finance	0.0889	0.1247	0.1101	RF
BAC	Finance	0.0789	0.1045	0.0871	RF
XOM	Energy	0.0675	0.0977	0.0789	RF
CVX	Energy	0.0694	0.0834	0.0826	RF

**Fig 3:**
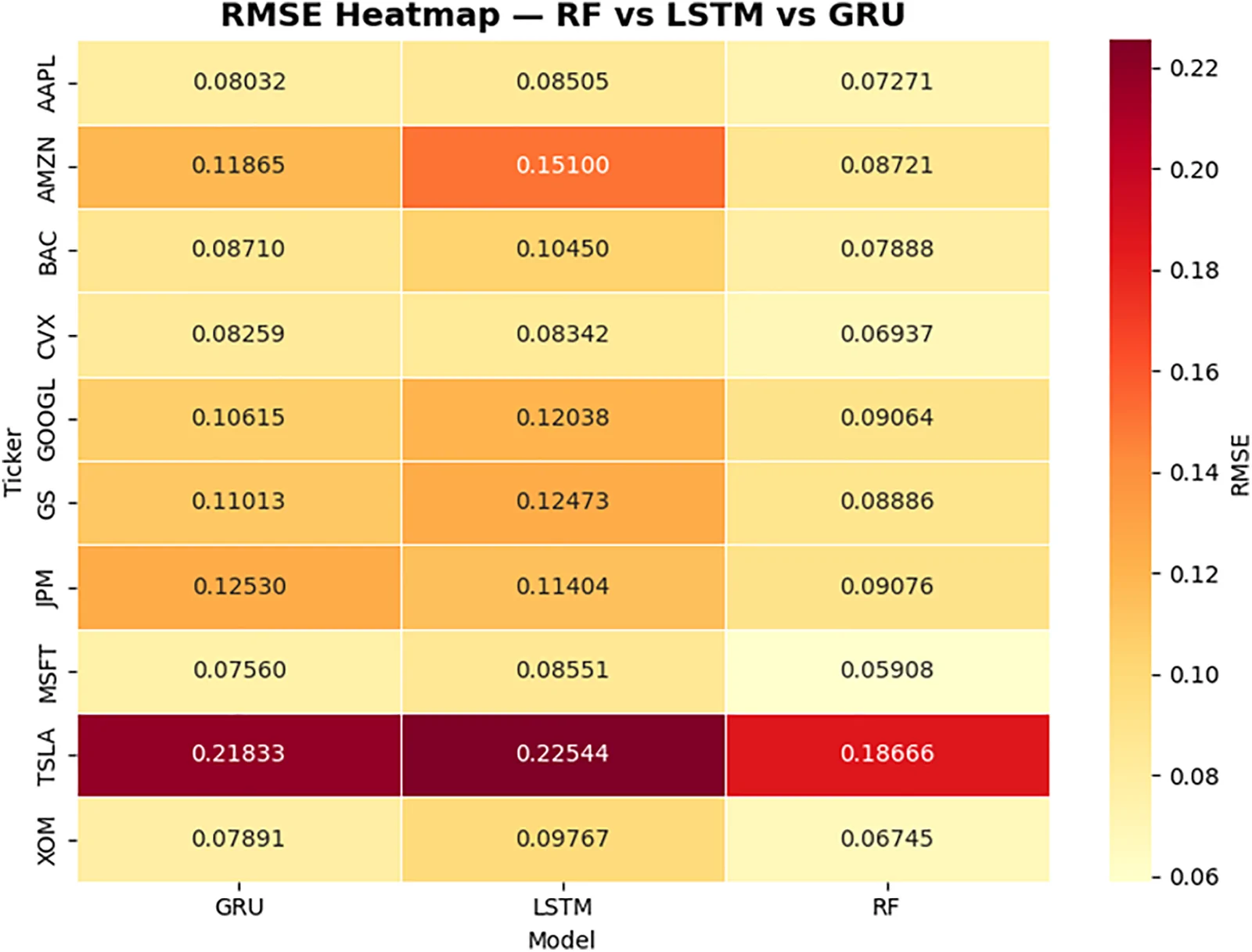
Heat map of all tickers and machine learning models displayed through RMSE (YlOrRd scale; dark grey represents highest error). Random forest consistently appears as the lightest (indicating lowest average RMSE) confirming it as the best performing ML model on the totality of tickers. TSLA dominated the RMSE for all machine learning models.

### C. Feature ablation study

The RF RMSE comparison in Fig 4 and Table 4 displays the difference between the returns-only baseline and the complete 13-feature set. The addition of realised volatility lags and volume results in reduced RMSE for 7 of the ten tickers, while AMZN (−3.2%), BAC (−6.9%), and XOM (−0.01%) show a slight increase in RMSE instead. The greatest reduction occurs at both MSFT (+11.6%) and AAPL (+7.2%), while TSLA has little to no improvement (+0.9%). The low improvement for TSLA indicates that its volatility is primarily driven by news flow rather than autocorrelation, while the negative results for AMZN, BAC, and XOM suggest that for some tickers the additional lagged features add noise rather than signal. The aggregate improvement across all tickers is 3.17%.

**Table 4:** Characteristic removal analysis: Predicting stock returns with only returns versus predicting stock returns with the complete set of features.

Ticker	Returns-Only RMSE	Full Features RMSE	RMSE Reduction (%)
AAPL	0.0783	0.0727	+7.2%
MSFT	0.0668	0.0591	+11.6%
GOOGL	0.0962	0.0906	+5.8%
AMZN	0.0845	0.0872	−3.2%
TSLA	0.1884	0.1867	+0.9%
JPM	0.0952	0.0908	+4.7%
GS	0.0949	0.0889	+6.3%
BAC	0.0738	0.0789	+6.9%
XOM	0.0674	0.0675	−0.01%
CVX	0.0732	0.0694	+5.2%
Mean	—	—	+3.17%

**Fig 4:**
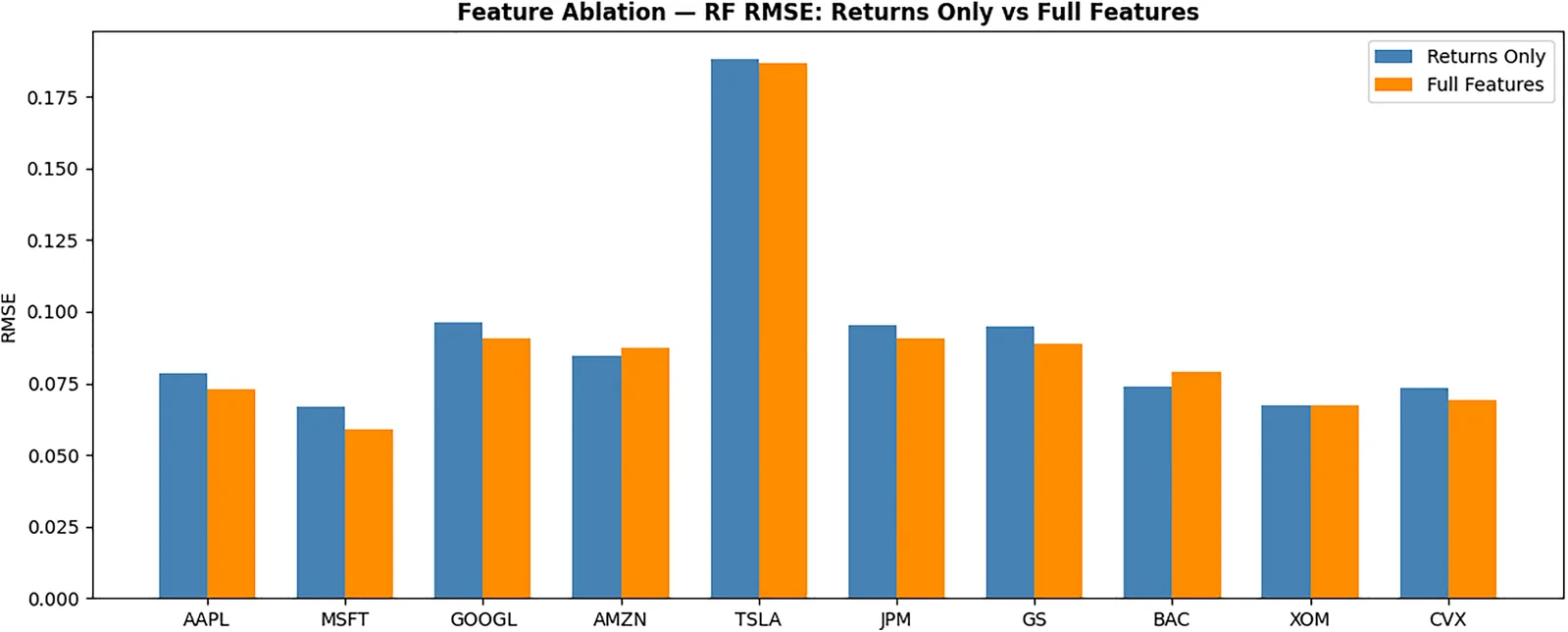
Comparison of the return-only baseline (blue) and all features (including RV delays and size) in terms of their ability to predict stock returns relative to each ticker (orange). The majority of tickers experienced a reduction in the root mean squared error when using the full set of features, especially Microsoft and Apple.

### D. Market regime analysis

Fig 5 illustrates the K-Means regime classifications for Apple Inc., created based on realised-volatility levels, where the stressed regime (red) was sparse but specifically identified true events of high risk such as the Q4 sell-off in 2018, the March/April 2020 COVID crash, and the exceptionally volatile market during the 2022 rate hikes. Table 5 provides a summary of the average root mean square error for each regime, pooled across tickers, with RMSE values in the “stressed” regime being approximately 3.7–4.3 times greater than in “calm” regimes across all models; these RMSE values illustrate the primary obstacle to accurately forecasting tail volatility. Random Forest retained its accuracy advantage over LSTM and GRU even in the stressed regime, though the absolute forecast error rises sharply for all three models under stress.

**Table 5:** Per-regime RMSE (mean across all tickers).

Regime	% of Days	RF RMSE	LSTM RMSE	GRU RMSE
Calm	65.8%	0.0776	0.1005	0.0907
Normal	29.6%	0.1079	0.1475	0.1356
Stressed	4.6%	0.2881	0.4292	0.3902

**Fig 5:**
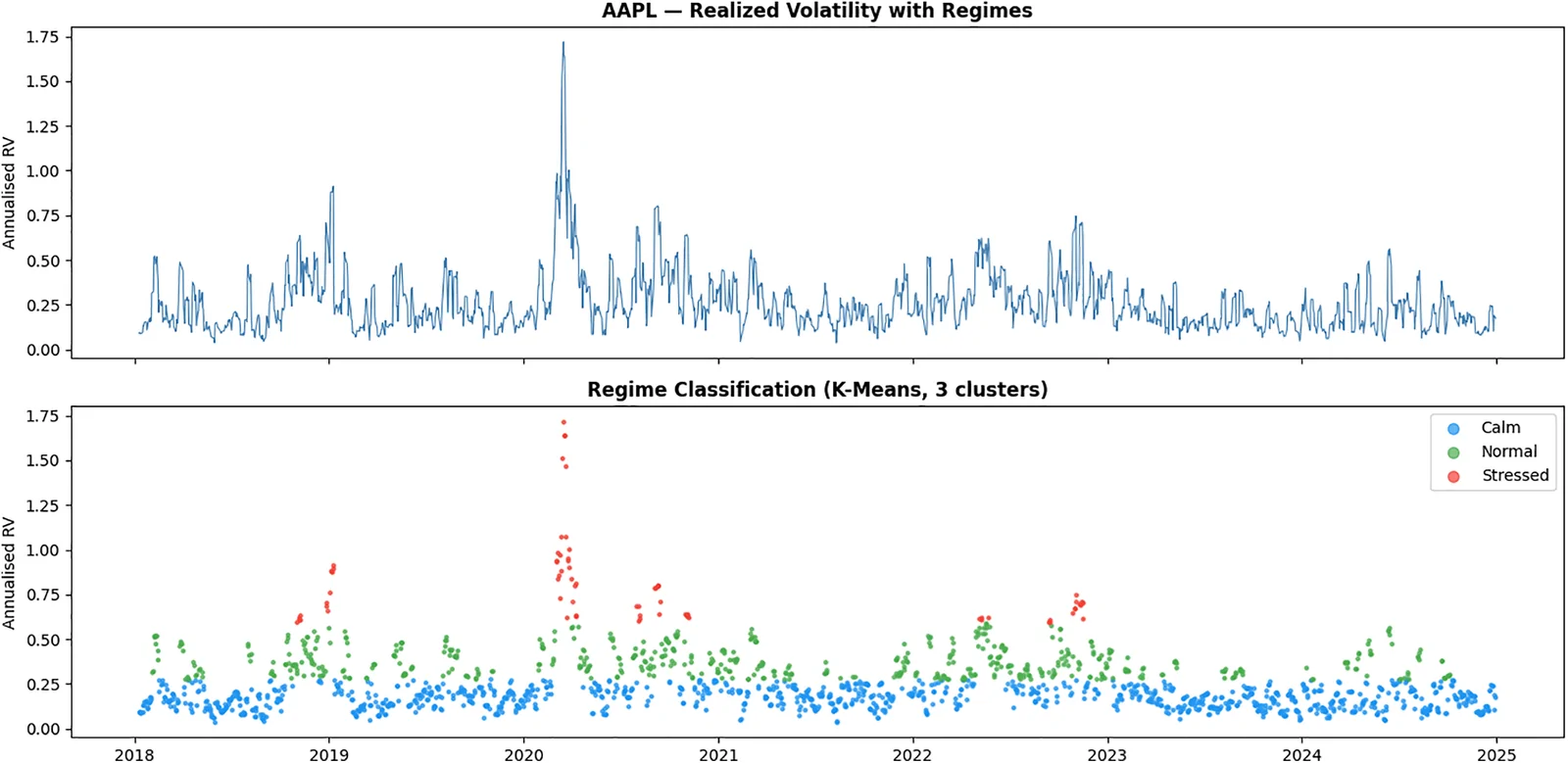
The market regime timeline for AAPL demonstrates continuous realized volatility. Realized volatility has been colour coded using K-Means to indicate the three market regimes (Calm — blue, 65.8% of days; Normal — green, 29.6%; and Stressed — red, 4.6%) and the timeline accurately reflects actual periods of stress in these three regimes.

### E. Computational efficiency

The time and memory benchmarks are shown in Fig 6 and detailed in Table 6. Random Forest has a ~ 7 × advantage in training speed vs. LSTM/GRU and uses <1 MB of memory vs. ~ 10 MB of memory consumed by deep learning models. Since RF also has a higher level of forecast accuracy than either LSTM or GRU, it is superior in terms of both accuracy and efficiency for this application and therefore comes to dominate the accuracy vs efficiency frontier for the application described.

**Table 6:** Computational efficiency benchmarks (mean across 10 tickers).

Model	Mean Train Time (s)	Peak Memory (MB)	Rank (Efficiency)
Random Forest	~5.2	< 1	1st
LSTM	~34.1	~10.3	3rd
GRU	~37.4	~10.1	2nd

**Fig 6:**
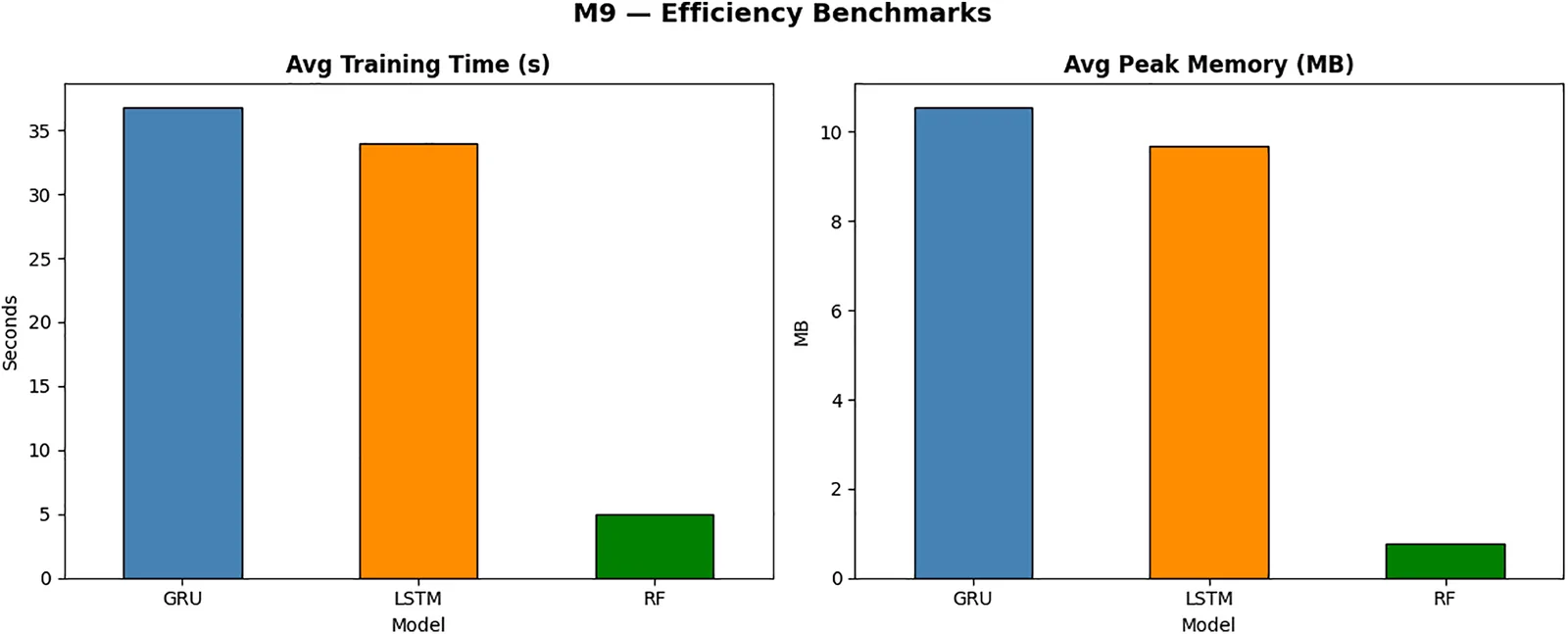
Computational efficiency benchmarking. Left: average training time per model in seconds; Right: average peak memory used in MB. Random Forest is about 7 × faster and 10 × more memory efficient than LSTM or GRU.

### F. Summary dashboard

Fig 7 presents the major results from each of the models on the 9-panel summary dashboard and demonstrate how the models compared on various measures: average errors (RMSE, MAE, QLIKE) by model, the last-100-day predictions for AAPL by model, the regime distributions for both markets and individual stocks by model, the ablation study comparisons by model and the time it takes to train the models. Random Forest (green bars) has the lowest average errors across all three measures. In contrast, LSTM (orange) is the worst performer on average across all three measures, especially in QLIKE, confirming the systematic underestimation of short-term volatility in its predictions. The prediction traces indicate that each of the models predicted the overall trend but differed by model as to how they responded to short-term spikes in volatility.

**Fig 7:**
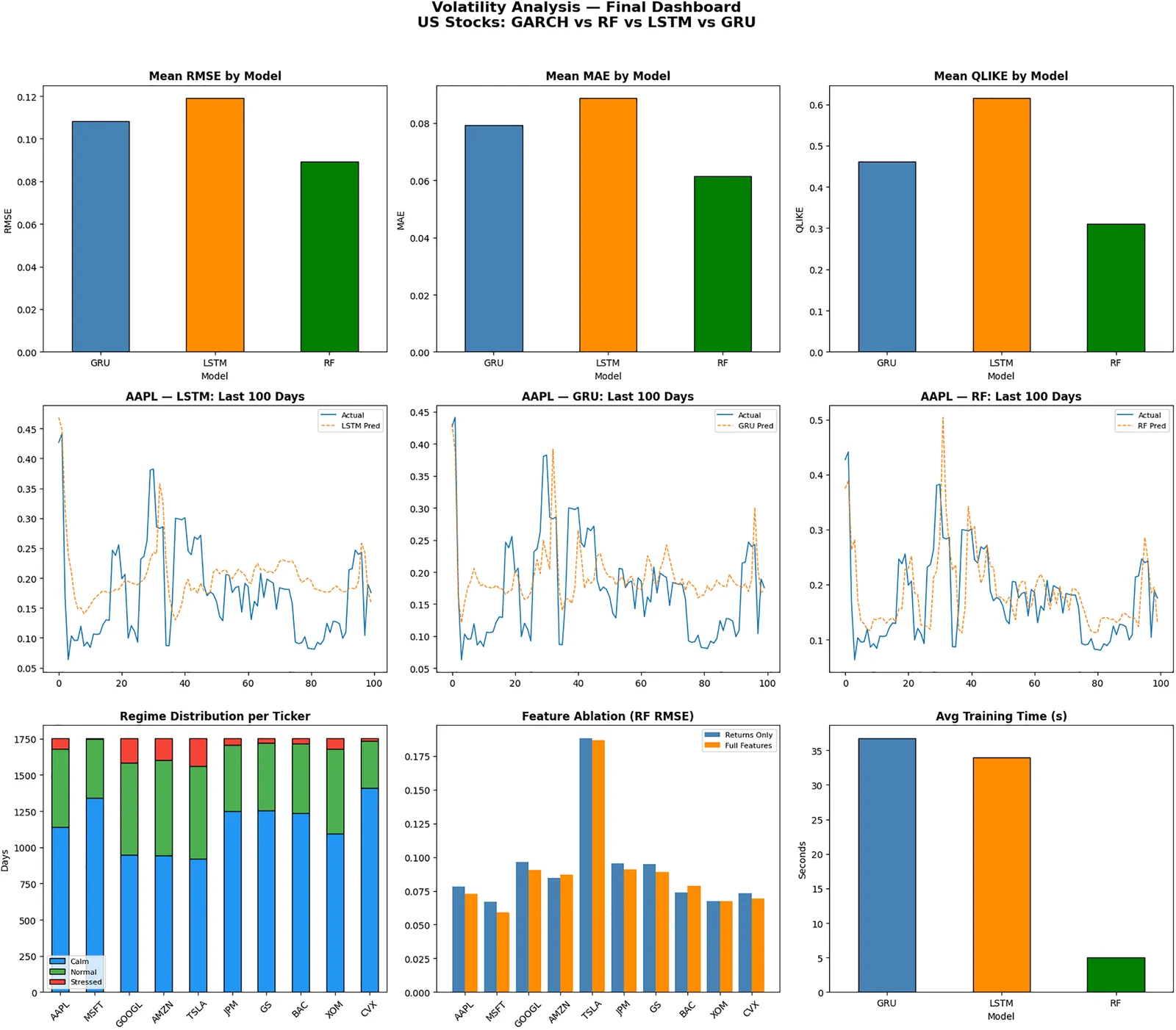
Summary dashboard – 4 different metrics shown on 9 panels. Row 1 – Mean RMSE, MAE and QLIKE (by model) indicates ‘Random Forest’ does consistently better than all other models. Row 2 – Comparison of predicted vs. actual prices for the last 100 days by model (i.e., LSTM, GRU and RF) for AAPL. Row 3 – Regime Distribution for each company, results of feature ablation and a comparison of the training time required by model for training each model.

## VII. Conclusion

This research paper provides a robust research study of forecasting stock-level volatility using various models such as GARCH, GJR-GARCH, Random Forest, LSTM, and GRU based on their respective performance from January 2018 through December 2024 [29]. In addition, this study was different than much of the published index-based volatility literature (particularly [9]), which forecasted the market direction of the S&P 500 by applying and benchmarking several different volatility indicators (CBOE volatility indices) to the S&P 500, but did not include direct applications of different forecasting models or benchmark model performance using individual stock-level volatility characteristics.

Key findings from this study include the following: (1) Random Forest produced the best forecasting error across the study with RMSE = 0.0892, MAE = 0.0614, QLIKE = 0.3105 compared to all other models used and was ~ 7 × faster (computationally) and ~10 × less memory intensive than any of the deep learning alternatives. (2) GRU produced better forecasting performance based on RMSE, MAE, and QLIKE than LSTM, despite GRU models having fewer parameters. (3) GARCH family models continued to provide competitive performance for value-at-risk (VaR) and expected shortfall (ES) calibration. (4) Lagged actual volatility and lagged actual volume provided meaningful and incremental additional predictive value (RMSE improvement of ~3.17%) and (5) All models presented significantly less accurate forecast performance during stress periods, highlighting the ongoing difficulty in predicting tail volatility.

Improvements for upcoming studies include adding high-frequency realised volatility indices, implied indicators from options, macroeconomic surprises, and new sentiment. The present findings could be generalized if the framework were extended to international equity markets and across different asset classes. Calibration error in predictive uncertainty intervals will serve as the follow-up building block; this will be the case for any risk management solution that employs Bayesian neural networks.
